# Coactivation of TLR2 and TLR8 in Primary Human Monocytes Triggers a Distinct Inflammatory Signaling Response

**DOI:** 10.3389/fphys.2018.00618

**Published:** 2018-05-29

**Authors:** Korbinian Bösl, Miriam Giambelluca, Markus Haug, Marit Bugge, Terje Espevik, Richard K. Kandasamy, Bjarte Bergstrøm

**Affiliations:** ^1^Centre of Molecular Inflammation Research, Department of Clinical and Molecular Medicine, Norwegian University of Science and Technology, Trondheim, Norway; ^2^Department of Infection, St. Olav’s University Hospital, Trondheim, Norway; ^3^Centre for Molecular Medicine Norway, Nordic EMBL Partnership, University of Oslo and Oslo University Hospital, Oslo, Norway

**Keywords:** innate immunity, Toll-like receptors, Toll-like receptor 2, Toll-like receptor 8, signaling, monocytes

## Abstract

Innate immune signaling is essential to mount a fast and specific immune response to pathogens. Monocytes and macrophages are essential cells in the early response in their capacity as ubiquitous phagocytic cells. They phagocytose microorganisms or damaged cells and sense pathogen/damage-associated molecular patterns (PAMPs/DAMPs) through innate receptors such as Toll-like receptors (TLRs). We investigated a phenomenon where co-signaling from TLR2 and TLR8 in human primary monocytes provides a distinct immune activation profile compared to signaling from either TLR alone. We compare gene signatures induced by either stimulus alone or together and show that co-signaling results in downstream differences in regulation of signaling and gene transcription. We demonstrate that these differences result in altered cytokine profiles between single and multi-receptor signaling, and show how it can influence both T-cell and neutrophil responses. The end response is tailored to combat extracellular pathogens, possibly by modifying the regulation of IFNβ and IL12-family cytokines.

## Introduction

Monocytes and macrophages constitute critical cell types in the innate immune response (Shi and Pamer, 2011). These cells are equipped with germline-encoded pattern recognition receptors/sensors (PRRs) that aid in the recognition of various microbial components from microbes termed pathogen-associated molecular patterns (PAMPs) and self-derived danger-associated molecular patterns (DAMPs) released by damaged cells (Liston and Masters, 2017). Depending on the specific receptor-PAMP/DAMP match and whether multiple PRRs are engaged, various downstream effectors/pathways are activated, which prepare the cells to fend of the invading agents by activating degradation pathways and relaying signals such as cytokines to further alert innate and adaptive immune cells in the surrounding tissues and at distal sites (Ginhoux and Jung, 2014).

Toll-like receptors (TLRs), a major subgroup of PRRs, are type 1 transmembrane proteins with ligand binding extracellular domains composed of leucine rich repeats and cytoplasmic intracellular signaling domains known as the Toll/IL-1 receptor (TIR) domains (Botos et al., 2011). Currently there are 10 TLRs described in humans and 12 in mice, for most their ligands have been identified. TLRs can be broadly divided into two groups, depending on the subcellular location where they encounter their specific ligands. In humans, TLR1, TLR2, TLR4, TLR5 and TLR6 encounter their specific ligands at the cell surface, while TLR3, TLR7, TLR8 and TLR9 bind to their ligands in endosomes (Kaisho and Akira, 2006; O’Neill et al., 2013). Upon recognition of PAMPs and DAMPs, TLRs recruit adaptor proteins containing TIR domains such as MyD88 or TRIF, which acts as initiators of signal transduction pathways culminating in the activation of interferon regulatory factors (IRFs), NF-κB, or MAP kinases regulating the expression of cytokines, chemokines and type I Interferons (IFNs) (Kurt-Jones et al., 2000; Honda et al., 2005; Kawasaki and Kawai, 2014). For instance, TLR2 recognizes a variety of components derived from Gram-positive bacteria and downstream signaling requires TIRAP/Mal as an adaptor, which acts through the MyD88-dependent pathway, inducing activation of NF-κB and MAPK family members (Oliveira-Nascimento et al., 2012). TLR2 signaling is also dependent on the formation of a heterodimer, either of TLR1 and TLR2 or of TLR2 and TLR6. These hetereodimers have different affinities for lipopeptide ligands, but utilize the same adaptor molecules and have common signaling pathways (Farhat et al., 2008). On the other hand, TLR8 signaling is exclusively mediated through MyD88 after recognition single-stranded RNA and short double-stranded RNA, making it an important PRR for viral pathogens. In addition, TLR8 induces type I IFNs through IRF5 and IRF7 activation (Cervantes et al., 2012). Post-translational modifications (PTMs) plays a crucial role in the activation and regulation of this complex response (Liu et al., 2016). PTMs such as phosphorylation have distinct patterns depending on the type of activation (Kandasamy et al., 2016).

Several studies have shown that responses to bacterial and viral pathogens are not exclusively dependent on activation of individual TLRs, but results from complex TLR-TLR interactions (Underhill, 2007; Delaloye et al., 2009; Slater et al., 2010; Negishi et al., 2012). Nevertheless, the effects of engagement of more than one innate immune receptor in close temporal proximity are not well studied. Recently, it was reported that RNA derivate from bacteria could also activate TLR8, and TLR2 could be able to suppress IFNβ production induced by TLR8 activation (Mancuso et al., 2009; Bergstrøm et al., 2015; Kruger et al., 2015; Tanji et al., 2015; Lai et al., 2016; Gidon et al., 2017). To further understand the molecular mechanisms behind TLR2 and TLR8 interaction we studied changes in signaling pathways, gene expression and cytokines production in primary human macrophages stimulated with TLR2 and TLR8 ligands alone or in combination. Our results show that while prolonged PTMs on transcription factors could explain amplitude differences between TLR2 and TLR8 gene induction and cytokine production, suppression of IFNβ has wide-ranging consequences in shaping the overall immune response. Surface TLRs suppressing IFN β production via the TLR8-IRF5 axis after recognition of bacterial RNA can therefore be an important mechanism in shaping the adaptive immune response to pathogenic Gram-positive bacteria.

## Materials and Methods

### Isolation of Leukocytes

Buffy coats and serum were obtained from healthy donors at the St. Olavs Hospital blood bank with approval by the Regional Committee for Medical and Health Research Ethics (REC Central, Norway, No. 2009/2245). Monocytes were isolated by Lymphoprep^TM^ (Axis-Shield) density gradient according to the manufacturer’s instructions. Peripheral blood mononuclear cells (PBMCs) were selected by plastic adherence for 1 h in 6-well plates in RPMI 1640 supplemented with 2 mmol/L L-glutamine (Sigma-Aldrich) and 10% human serum, washed three times with Hanks balanced salt solution (Sigma-Aldrich) and rested for 1 h before stimulation. At specified time points cells were harvested and the cell pellet was stored at -80°C for RNA isolation or protein analysis. Supernatants were collected and stored at -80°C.

Purity of the isolated monocytes was validated using Flow Cytometry. Adhered monocytes were detached using Accutase (Sigma) solution (15 min, 4°C) and transferred to flow-tubes. Dead cells stained with Fixable Viability Dye eFluor780 (eBioscience). Fc receptor binding inhibitor (eBioscience) was used to block non-specific Fc receptor binding of antibodies. Cells were subsequently stained with fluorescence-labeled monoclonal antibodies to CD14 (PE/Cy7, BioLegend), CD11b (PE, BioLegend), CD3 (Brilliant Violet780, BioLegend), CD19 (Brilliant Violet510, BioLegend), CD56 (APC, eBioscience), CD303 (PerCP/Cy5.5, BioLegend). Flow-cytometry was performed on a BD LSRII flow-cytometer (BD Biosciences) and samples analyzed using FlowJo software (FlowJo, LLC).

A population of >85% CD14+/CD11b+ cells was observed, while surface markers for contaminating cell populations (CD3+, CD19+, CD56+, or CD303+) were detected with ≤0.6% each (**Supplementary Figure S1A**). mRNA expression of markers for non-monocytic cells types in all samples used for the Array-based RNA expression analysis were found to be low (**Supplementary Figure S1B**).

For viability analysis, monocytes were stimulated with FSL-1 (100 ng/ml) or CL075 (1 μg/ml) or both, unstimulated cells were used as a control. After 3 h of stimulation, calcein-AM (1 μg/ml, Invitrogen) and propidium iodide (1 μg/ml, Sigma) were added to stain viable and dead cells respectively. Subsequently, monocytes were detached and calcein-AM and propidium iodide fluorescence quantified by flow-cytometry on a BD LSRII flow-cytometer (BD Biosciences); samples analysis using FlowJo software (FlowJo, LLC) (**Supplementary Figure S2A**). We also performed microscopy analysis of monocytes to test the viability using the EVOS system (Thermo Fisher) (**Supplementary Figure S2B**).

Untouched human primary CD4^+^ T cells were isolated from PBMCs by negative selection using CD4^+^ T Cell Isolation Kit (Miltenyi Biotec) according to the manufacturer’s instructions. Purity of CD4^+^ T cells was assessed by flow cytometry with anti-CD4 (Alexa Fluor 700, eBioscience) and anti-CD3 [Brilliant Violet (BV) 785, BioLegend] antibody staining. Frequencies of contaminating cells were measured by antibody staining for CD8 (BV605, BioLegend), CD14 (PE/Cy7, eBioscience), and CD11c (PE, eBioscience). CD3 + CD4 + T cell purity was >95%.

Human neutrophils obtained from fresh peripheral blood drawn by venipuncture from healthy volunteers (approval by the Regional Committee for Medical and Health Research Ethics REC Central, Norway, No. 2009/2245) were isolated by double gradient centrifugation. 20 mL of heparinized blood were transferred to sterile polypropylene conical tubes containing a double gradient separation composed of equal volumes (10 mL) of Polymorphoprep (density, 1.113 g / mL) and Lymphoprep (density, 1.077 g / mL). Following centrifugation at 400 × *g* for 30 min, the layer of polymorphonuclear (PMN) cells was aspirated and contaminating erythrocytes were removed by 20 s of hypotonic lysis. Neutrophils were diluted in RPMI 1640 + 10% FCS to obtain a final cell concentration of 10^6^ cells / mL, with the purity and viability of neutrophils equal to or more than 95 and 98%, respectively.

### Ligands and Antibodies

TLR8 ligand CL075 (used at 1 μg/mL) and TLR2 ligand FSL-1 (used at 100 ng/mL) were purchased from Invivogen. Antibodies used for Western blots in this study were: anti-phospho p38 (Cell Signaling Technology, No. 9211), anti-phospho JNK (Cell Signaling Technology, No. 4668), anti-phospho ERK1/2 (Cell Signaling Technology, No. 4370), p38 (Cell Signaling Technology, No. 9212), JNK (Cell Signaling Technology, No. 9252), ERK1/2 (Cell Signaling Technology, No. 4695), and GAPDH (Abcam, No. ab8245).

### Western Blotting

Cells were lysed in cell lysis buffer (Bergstrøm et al., 2015), centrifuged. The cleared lysates were then mixed with 4x LDS loading buffer (Life Technologies) and PAGE was performed with the NuPAGE system (Life Technologies) as per the manufacturer’s recommendations. Immunoblotting was performed with the iBlot system (Life Technologies) as per the manufacturer’s recommendations. Membranes were blocked with 5% BSA in Tris-buffered saline (TBS, pH 7,4) and incubated with primary antibodies overnight at 4°C. Membranes were then washed in TBS with 0.5% tween-20 (TBS-T), incubated with HRP-conjugated secondary antibodies (Dako, No. P0399 and No. P0447) for 1 h at room temperature. Blots were developed with SuperSignal West Femto (Pierce) and imagined on a Li-Cor Odyssey system.

### Multiplexed Cytokine Profiling

Supernatants were diluted 1:20 and analyzed according to the manufacturer’s protocol using the Human ProcartaPlex 34-plex panel 1A (ThermoFischer, No. EPX340-12167-901) on a Bio-Plex 200 instrument (BioRad).

### Array-Based RNA Expression Analysis

Gene expression was analyzed with an Illumina HT-12 v4 bead array as per the manufacturer’s instruction and performed at the Genomics Core Facility at NTNU. RNA isolation was performed using RNeasy mini kit (Qiagen) according to the manufacturer’s instructions. RNA integrity was examined by Bioanalyzer (Agilent). Hybridization to a HumanHT-12 v4.0 bead array was performed by the Genomics Core Facility (Department of Cancer Research and Molecular Medicine, NTNU). The Data was preprocessed using GenomeStudio v1.9.0 and imported to R/Bioconductor v3.3.2/3.4 (Huber et al., 2015). Probes with detection *p*-value > 0.05 and probes with reported unspecific binding were excluded. The expression data was background corrected using negative control probes and quantile normalized in limma v3.32.9 (Ritchie et al., 2015).

Inter-donor variation was included as one of the parameters for the linear model used in the analysis. Genes showing an expression log2-fold > 2.5 and FDR < 0.05 compared to the unstimulated samples for each time-point were considered differential expressed. Differential expressed genes were clustered using Manhattan distance matrix calculation and divided into 8 clusters for further analysis. Gene Ontology analysis was performed with clusterProfiler v3.4.4 (Yu et al., 2012), the selection of Gene Ontology Terms for visualization was curated manually. Raw data can be accessed from ArrayExpress database using the accession number E-MTAB-6222.

### T Cell Differentiation Assays

Human primary CD4^+^ T cells (0.5 × 10^6^ cells/well) were activated with anti-CD3 (plate-coated, 5 μg/mL, eBioscience) and anti-CD28 (1 μg/mL, eBioscience) on 96-well plates. CD4^+^ T cells were differentiated for 2 – 8 days at 37°C in 100 μL RPMI 1640 medium (Sigma) supplemented with 10% A+ serum and 100 μL supernatants from TLR2/TLR8 stimulated monocytes stimulated with TLR ligands. CD4^+^ T cell effector cytokine production was analyzed 48 h post activation and on day 8 after 6 h short-term re-stimulation with Cell Stimulation Cocktail (eBioscience). Protein Transport Inhibitor Cocktail (eBioscience) was added for the last 4 h before harvest of the cells. Cell were stained with Fixable Viability Dye eFluor 780 (eBioscience) and surface-stained with fluorescent antibodies to CD3 (BV 785, BioLegend), CD4 (Alexa Fluor 700, eBioscience) before fixation and permeabilization (FOXP3 buffer set, BD Biosciences). Staining for intracellular cytokine production was performed with fluorescent antibodies to IFN-γ (FITC, Miltenyi Biotec), IL-17 (BV 510, BioLegend), IL-2 (PE/Cy7, eBioscience), IL-10 (APC, eBioscience) IL-4 (PE-Vio615, Miltenyi Biotec) and TNF-α (BV421, BioLegend) and Multicolour flow cytometry was performed on a BD LSRII flow cytometer and analyzed with FlowJo software (FlowJo, LLC).

### Neutrophil Migration Assay

Chemotaxis was measured as described previously (Gilbert et al., 2003), with some modifications. Briefly, neutrophils were resuspended in RPMI 1640 and 10% FBS at 10^6^ cells/mL and were preincubated with 5 μg/mL CellTracker Deep Red (Molecular Probes, Eugene, OR, United States). After 30 min incubation at 37°C in the dark, cells were washed twice and re-suspended in RPMI/FCS at 3 × 10^6^ cells/mL. Neutrophil migration was monitored using a 96-well chemoTX disposable chemotaxis system (NeuroProbe, Gaithersburg, MD, United States). The bottom wells were filled with 31 μL of macrophage supernatant or a dilution of the chemotactic agent. The polycarbonate filter was positioned on the plate, and neutrophils (25 μL) were seeded on the filter and allowed to migrate for 1 h at 37°C in the presence of 5% CO2 in the dark. Non-migrated cells were gently removed by wiping the filters with a tissue. The fluorescence of the cells in the filters was measured with a microplate fluorescence reader (Excitation and emission wavelengths, 630 and 660 nm, respectively). Fluorescence was transformed to numbers of neutrophils based on a standard curve generated by seeding known numbers of neutrophils in the bottom of the chamber. The results were expressed as percentage of migrated neutrophils.

### Statistical Analysis

Statistical analysis was done in R v3.3.2. After testing for normal distribution a paired Wilcox test was performed. Significance levels are indicated as followed: ^∗^*p* < 0.05, ^∗∗^*p* < 0.01, ^∗∗∗^*p* < 0.001, ^∗∗∗∗^*p* < 0.0001.

## Results

### Transcriptome Analysis of Primary Human Monocytes Stimulated With TLR2/8 Ligands

**FIGURE 1:**
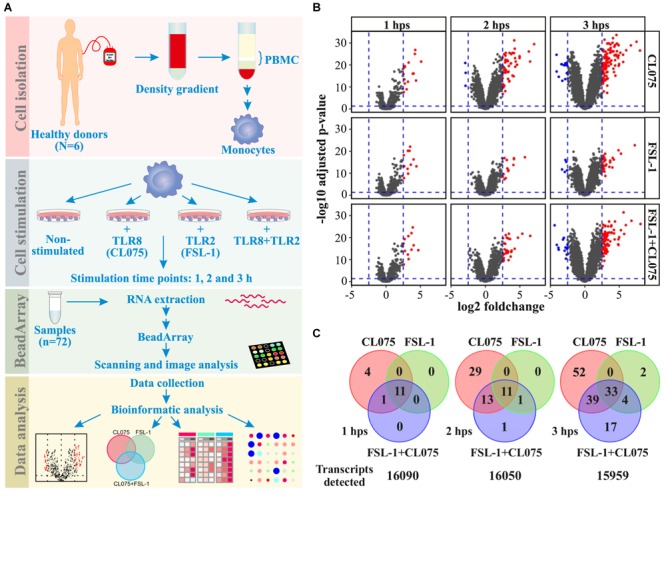
Distribution of differentially regulated genes after TLR8, TLR2 or co-stimulation in monocytes. **(A)** Human primary monocytes (*n* = 6) were stimulated with a TLR8 ligand (CL075, 1 μg/mL), a TLR2 ligand (FSL1, 100 ng/ml) or both and cells were lysed and RNA extracted after 1, 2, and 3 h. **(B)** Transcriptome analysis was performed using Illumina HT-12 v4 bead array. Corresponding volcano plots shows that TLR8 stimulation gives a larger magnitude of differentially expressed genes than TLR2 stimulation alone or co-stimulation compared to non-stimulated samples at the same time-point. **(C)** A common core set of inflammatory genes are induced, but TLR8 stimulation induce more unique genes at later time points than TLR2 stimulation. Co-stimulation induces more genes than TLR2 but less than TLR8 alone, with a significant subset shared only with TLR8 stimulation.

We performed bead array-based transcriptomics analysis to identify genes induced by the stimulation or co-stimulation of TLR2 and TLR8. We used FSL-1 (Pam2CGDPKHPKSF - a synthetic lipopeptide) as a TLR2/TLR6 agonist; and CL075 (a thiazoloquinolone derivative) as TLR8 agonist. Primary human monocytes from six different donors were isolated and stimulated with FSL-1 and/or CL075 for 1, 2, and 3 h; and were subjected to transcriptome analysis (**Figure 1A** and **Supplementary Figure S3**) to quantify the expression of 16,106 genes. Applying a log2(fold-change) of 2.5 and adjusted *p*-value < 0.05 cutoff, we identified 63, 194, and 132 genes differentially regulated at one or more time-points by FSL-1, CL075 and co-stimulation, respectively (**Figure 1B**). We found classic inflammatory genes (Turner et al., 2014) like TNFα, IL-6, CCL and CXCL family of cytokines highly upregulated by both TLR2 and TLR8 stimulation – alone and together. One of the highly induced genes specific to TLR8 stimulation at 1 h was Interferon β, as expected. The distribution of induced genes at the earliest time point (1 h) showed a large overlap between stimuli, where 11 out of 16 genes are induced by all stimuli alone or together. This distribution changed at later time points, where TLR8 stimuli induced an additional set of genes that were not induced by TLR2 (**Figure 1C**). Co-stimulation falls between TLR2 and TLR8 stimulation alone, suggesting that TLR2 signaling partially, but not completely, regulates TLR8 signaling. After 3 h of stimulation, the core response induced by all stimuli effectively triples compared to the earlier time points, consistent with the start of a secondary response. Together these results show that both stimuli share a core early response while TLR8 induced an additional set of genes as a separate and/or secondary response, and that upon co-stimulation TLR2 signaling modulates the TLR8 response. Macrophage differentiation markers such as CCR5, FCGR1 and CD68 are not affected during the tested timespan, while CD14 and MERTK show a down-regulation and CD40 and CD71 (TFRC) show an up-regulation from after 2 h, sharing the same pattern as the general inflammation markers (**Supplementary Figure S4**).

### Cluster Analysis of the Transcriptome Data Reveals Ligand Specific Transcriptional Activation

**FIGURE 2:**
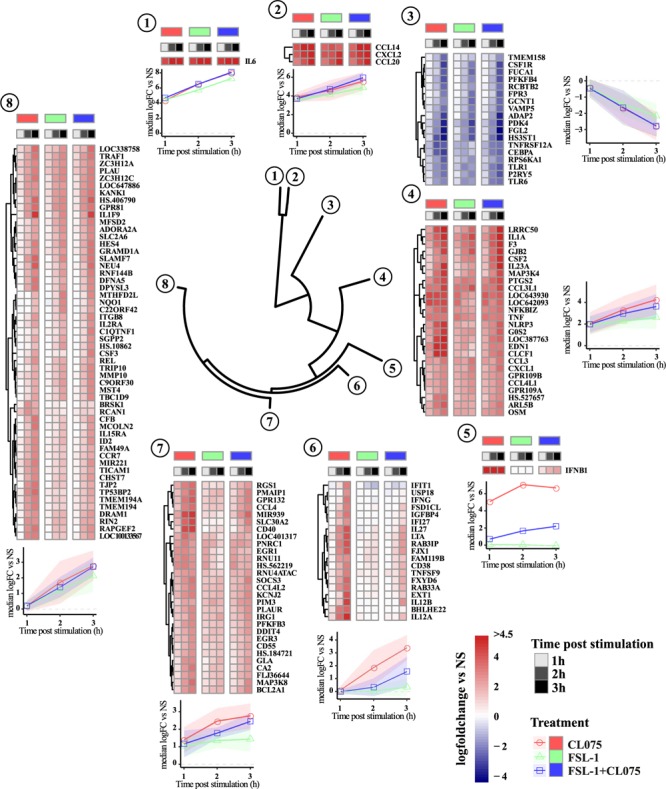
Clustering and magnitude of differentially regulated genes in monocytes. Gene transcript expression changes compared to non-stimulated samples were calculated as log fold change and hierarchical clustered using Manhattan distance matrix calculation and divided into 8 clusters. Clustering of the dataset positions IFNβ in a separate cluster. Genes in cluster 6–8 are induced later than genes in cluster 1, 2, and 4. Cluster 6 shows a clear pattern of being dominated by TLR8 stimulation with expression of several genes described as IFN-induced genes significantly reduced by co-stimulation. Overall TLR8 clearly induces a stronger response than TLR2, again with co-stimulation falling between the two responses alone.

To investigate the temporal and ligand-specific activation profile, we carried out clustering analysis of all the 151 differentially regulated genes (**Figure 2** and **Supplementary Table S1**). This segregated the differentially regulated genes into 8 different clusters. Genes in cluster 6–8 are induced at later time-points, hence being indirect TLR signaling targets. Cluster 1, 2, and 4 included the upregulated classic inflammatory genes such as IL-6, CCL14, CXCL1, CXCL2, CCL20, TNFα and PTGS2, which were common to TLR2/8 stimulation and co-stimulation. Cluster 3 included 18 downregulated genes that were common to both TLR2/8 stimulation as well as co-stimulation. Clusters 5 and 6 included differentially upregulated genes specific to TLR8 stimulation. Interferon β was the lone gene in cluster 5 with a unique expression profile while cluster 6 included several interferon-induced genes such as IFIT1 and IFI27 (Rasmussen et al., 1993; Guiducci et al., 2013). Clusters 7 and 8 included several upregulated genes that were common to TLR2/8 stimulation and co-stimulation, though we see a stronger upregulation by TLR8 stimulation. Altogether, this analysis shows that there is a common and ligand specific transcriptional activation with a unique mechanism for regulation of interferon β as reported earlier (Bergstrøm et al., 2015).

We performed gene-ontology enrichment analysis to gain insights into the biological processes and signaling pathways that are affected by these differentially regulated genes. We identified several biological processes (**Supplementary Table S2**) that were significantly enriched (adjusted *p*-value < 0.05) such as regulation of cytokine production, JAK-STAT signaling, NFκB signaling, cell chemotaxis, regulation of MAPK signaling, and regulation of adaptive immune response (**Supplementary Figure S5**). MAPK signaling has been shown to be essential for transcription of specific anti-inflammatory genes (Gottschalk et al., 2016). Corroborating with this data, our gene ontology analysis also pinpoints the possible role of three canonical MAP kinases p38, ERK1/2 and JNK.

### TLR2/8 Co-stimulation Affects the Phosphorylation Dynamics of MAPK Signaling

**FIGURE 3:**
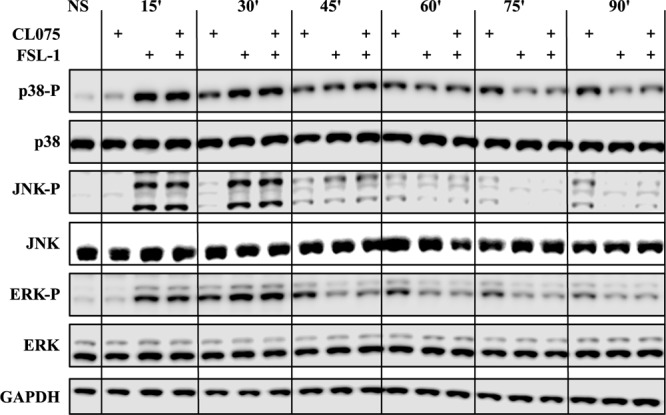
Co-stimulation affects the MAP kinase phosphorylation cascades. Human primary monocytes (representative donor shown) were stimulated as earlier described and protein lysates were collected in 15-min intervals after stimulation and evaluated for the phosphorylation status of the MAP kinases p38, ERK1/2 and JNK, as phosphorylation of these kinases are indicative of activation. TLR2 stimulation shows a rapid and strong phosphorylation of all three kinases which is also evident for co-stimulation. The kinases are then de-phosphorylated after about 45 min. TLR8 shows a slower but lasting phosphorylation pattern of these kinases. Here co-stimulation is dominated by the de-phosphorylation induced by TLR2.

MAPK signaling was one of the enriched biological processes regulated by the differentially expressed genes and it has been shown that TLR2 regulates MAPK signaling differently from TLR13 which is described as a murine TLR8 homolog (Choo et al., 2017). We probed phosphorylated forms of p38 (T180/Y182), JNK (T183/Y185) and ERK (ERK1/2-T202/Y204) in addition to their total protein levels by western blot (**Figure 3**). These phosphorylated forms are the functionally active versions that in turn activate various transcription factors such as AP-1 complexes. Upon TLR2 stimulation, the phosphorylation levels of p38, JNK and ERK peaks between 15 and 30 min and then starts to reduce. This suggests that TLR2 induces a feedback inhibition that reduces the level of MAPK signaling activity. TLR8 stimulation induces a lesser degree of MAPK phosphorylation at early time points but is prolonged compared to TLR2 signaling and does not show the same dephosphorylation kinetics as TLR2 signaling. Co-stimulation of TLR2 and TLR8 fits closely to the TLR2 signaling profile, showing that the same feedback mechanism limiting MAPK signaling after TLR2 stimulation also limits signaling induced by TLR8.

### Cytokine Profiling Shows Commonality and Specificity of TLR2/8 and Co-stimulation

**FIGURE 4:**
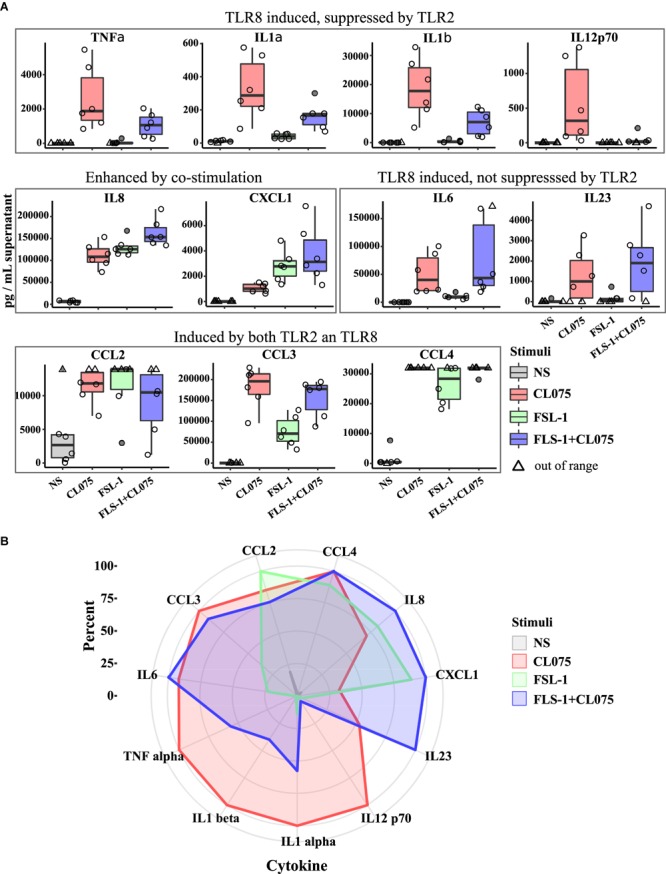
Differential cytokine secretion after TLR8, TLR2, and co-stimulation. Human primary monocytes were stimulated as earlier and supernatant was collected after 6 h and analyzed by bioassay (*n* = 6). **(A)** The differences in gene expression presented in **Figure 2** is also evident in cytokine production and release, but while TLR2 is less potent to drive gene expression it is as or more potent at releasing some cytokines. Co-stimulation also notably induce higher amount of certain cytokines, especially those involved in neutrophil stimulation and attraction such as IL8 and IL23A. **(B)** Plotting the measured cytokine levels in a radar plot gives three overlapping but distinct cytokine profiles of the different stimulation status. Significance values are provided in **Supplementary Table S3**.

The transcriptomics analysis showed transcriptional upregulation of several cytokines. We performed multiplexed cytokine profiling to measure the amount of secreted cytokines after 6 h of TLR2/8 activation and found 11 different cytokines that were significantly regulated (**Figure 4A**). We could divide these cytokines into four different groups: (1) induced by TLR8 but suppressed by TLR2 activation - TNFα, IL-1α, IL-1β, and IL12p70; (2) induced by both TLR2/8 stimulation and enhanced by co-stimulation – IL-8 and CXCL1; (3) induced by TLR8 and not suppressed by TLR2 stimulation – IL-6 and IL-23; and (4) induced by all and not affected by co-stimulation – CCL2, CCL3, and CCL4. To get a more global view of this cytokine response to TLR2/8 activation, we visualized the relative levels of cytokines in percentage as a spider plot (**Figure 4B**). We observed that TLR2 gives a limited response with a strong chemokine component related to the recruitment of phagocytosing cells while TLR8 signaling induces a mixed Th1/Th17-activating profile with a strong induction of IL12p70 and IL-1β. Co-stimulation enhances the release of neutrophil-recruiting cytokines such as IL-8 and CXCL2, but also provide a shift from IL12p70 to IL23 induction that would enable Th17 activation on the expense of Th1 activation. Thus, co-stimulation of monocytes should be able to induce both an acute innate and an adaptive neutrophil-based response through neutrophil-attracting chemokines and the Th17-polarizing cytokine IL-23.

### TLR2/8 Co-stimulation Modulates Neutrophil Migration and T Cell Differentiation

**FIGURE 5:**
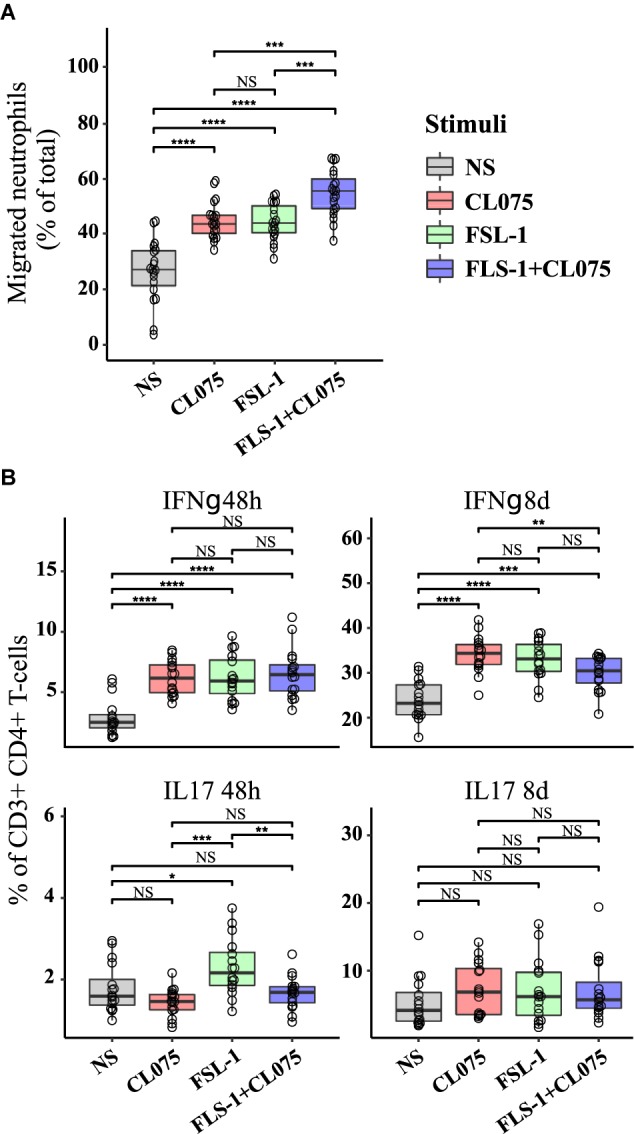
Functional effects on immune activation *in vitro* of differential cytokine regulation. **(A)** Human neutrophils were isolated from the blood of healthy donors (*n* = 3), loaded with CellTracker Deep Red and placed on a polycarbonate filter above a well containing supernatants from stimulated human monocytes (*n* = 8) or RPMI. Neutrophil migration was measured after 60 min incubation. While TLR8 and TLR2 stimulation alone increased migration in a similar manner, co-stimulation was significantly increased compared to either stimuli alone as predicted. **(B)** Human primary CD4^+^ T cells were differentiated with supernatants from non-stimulated and stimulated monocytes (*n* = 8). IL17-positive T cells, indicative of a Th17 phenotype was increased by TLR2 stimuli after 48 h, while IFNγ-positive T cells, indicative of a Th1 phenotype was reduced by co-stimulation after 8 days. ^∗^*p* < 0.05, ^∗∗^*p* < 0.01, ^∗∗∗^*p* < 0.001, ^∗∗∗∗^*p* < 0.0001 paired Wilcox test.

In order to verify how co-stimulation can affect T cell and neutrophil response, we implemented two assays with supernatants from stimulated monocytes: (1) a neutrophil migration assay; and (2) a T-cell differentiation assay. The supernatant of primary human monocytes stimulated with TLR2 and TLR8 ligands, alone or in combination was used in a migration assay with peripheral human neutrophils from healthy donors. We found that migration was increased in neutrophils migrating toward the supernatant of TLR2 and TLR8-stimulated monocytes as compared to those migrating toward supernatant from unstimulated monocytes. The chemotaxis of human neutrophils was significantly higher with supernatants of TLR2/8 co-stimulated monocytes (**Figure 5A**). This effect is specifically attributed to the supernatants from the stimulated monocytes since the TLR2/8 ligands themselves does not have any significant effect on the neutrophil migration (**Supplementary Figure S6**).

Primary human CD4^+^ T cells were isolated by negative selection and treated with a conditioned medium containing the supernatants derived from TLR2/8 stimulated monocytes. CD4^+^ effector T cell differentiation was assessed by the percentage of IFNγ (Th1 subset) and IL-17 (Th17 subset) positive cells after 48 h and 8 days (**Figure 5B**). No significant difference was noted in the Th1 subset after 48 h of differentiation, but after 8 days a small but significant difference was noted where Th1 differentiation was suppressed by co-stimulation compared to TLR8 stimulation alone. Supernatant from TLR2-stimulated monocytes were more efficient in initiating Th17 differentiation as seen after 48h compared to supernatants from TLR8 and co-stimulated monocytes which showed a small inhibitory effect on Th17 differentiation. These differences were not evident after 8 days where all stimuli induced a robust Th17 differentiation. Together these experiments indicate that differential signaling induced by co-activation of TLR8 and TLR2 can have specific and emergent effects on the immune response.

## Discussion

TLR8 is one of the lesser-studied TLRs, with a restricted expression pattern and an unresponsive murine homolog (Guiducci et al., 2013). It was previously established as an antiviral receptor based on its location in the endosome and its ability to induce IFNβ. More recently, TLR8 has been reported to be a general sensor of RNA breakdown products (Kruger et al., 2015) and shown to be important also for the recognition of bacterial infections (Cervantes et al., 2013; Bergstrøm et al., 2015; Eigenbrod et al., 2015). The role of IFNβ production in response to bacterial infections is, however, controversial and depending on the nature of the pathogen and the host IFNβ can be both beneficial and detrimental in the clearance of bacterial infections (Boxx and Cheng, 2016).

TLR2 is a sensor of cell wall products and generally considered to be a sensor for Gram-positive bacterial infections via its ability to detect lipoteichoic acid from bacterial cell walls (Oliveira-Nascimento et al., 2012). TLR2 also has the ability to suppress IFNβ induction by TLR8 if signaling is initiated simultaneously or sequentially in short order by an unknown mechanism that is IRF5 dependent (Bergstrøm et al., 2015). We initially set out to identify possible other targets of this mechanism and were surprised when IFNβ appeared as a lone differentially regulated cluster (**Figure 2**). Although we cannot exclude that other differentially expressed target genes such as IL-6 (Steinhagen et al., 2013; Yasuda et al., 2013) or TNF (Krausgruber et al., 2010) seen in cluster 4 are also regulated by IRF5 (Steinhagen et al., 2013), regulation of mRNA stability could be a likely candidate to explain the differences when we take into account the differences seen for these genes on the protein secretion level (Deleault et al., 2008; Iwasaki et al., 2011). Several inflammatory response genes are reported to be regulated on the mRNA stability level, e.g., by Regnase-1 in an IKKβ-dependent loop (Iwasaki et al., 2011) or by Tristetraprolin (TTP) which again is regulated via a p38 MAPK-dependent signaling loop (Kratochvill et al., 2011).

It is well established that both TLR8 and TLR2 signaling is dependent on activation of MAPK pathways (Arthur and Ley, 2013) and that different TLRs propagate the MAPK-dependent signal with different amplitudes and temporalities (Choo et al., 2017). Thus, it is not surprising that MAPK signaling appears to be a differentially regulated process in the GO analysis and together with the reports on MAPK-dependent mRNA stabilization we found these differences to be interesting enough to study further. The fact that upon simultaneous stimulation the TLR2 signal (high amplitude, short duration) will override the TLR8 signal (slower, less amplitude, longer duration) has not been shown before. These properties are as important as the presence of the signal by itself (Atay and Skotheim, 2017). It is not clear how this affects TLR8/2 signaling, but it is evident that TLR2 signaling induces a potential feedback inhibitory loop that reduce the intensity of the signal, probably via the activation of one or several phosphatases (Kondoh and Nishida, 2007). This feedback loop should also affect mRNA stability and could at least partially explain the higher cytokine secreted levels seen by TLR8 signaling compared to TLR2 signaling. Since IRF5 is also activated by phosphorylation (Chang Foreman et al., 2012), suppression of IRF5 activation could also be a by-stander effect of MAPK phosphatase activation. Several phosphatases including dual specificity phosphatases (DUSP) have been reported to function as MAPK phosphatases that control inflammatory response (Chu et al., 1996; Zhang et al., 2004; Zhao et al., 2005; Lang et al., 2006). Although several phosphatases including dual specificity phosphatases such as DUSP1, DUSP2, DUSP4, DUSP5, DUSP9, DUSP10 and DUSP16 were upregulated (**Supplementary Figure S7**), we didn’t find any difference between mRNA expression of these phosphatases in TLR8 stimulated and co-stimulated cells.

The ramifications of IFNβ suppression can be clearly observed in cluster 6 and 7, as TLR8-induced IFNβ autocrine or paracrine signaling induces a number of genes that are not or barely induced by TLR2 and are severely suppressed in co-stimulation. The functional difference between these stimuli is shown on the cytokine secretion level, where TLR8, TLR2 and co-stimulation induce overlapping but discrete cytokine profiles. TLR2 induce a limited but effective cytokine response alone, tailored to attract neutrophils. TLR8 induce a broader response partially dependent on IFNβ, particularly concerning IL-12-family cytokines IL-12p35 and IL-12p40. Together these form a heterodimeric cytokine named IL12-p70 which is a known activator of Th1 differentiation processes. Co-stimulation shifts this focus over to induction of IL-23p19 on the expense of IL12-p35. The resulting heterodimer of IL-12p40 and IL-23p19 is known as IL-23, a powerful enhancer of Th17 differentiation. These findings are in concordance with earlier reports that place IRF5 in a central position in inducing Th1/Th17-polarizing cytokines (Krausgruber et al., 2011), but also show that when IRF5 activation is suppressed it only affects the Th1 component of this response but not the Th17 component (**Figure 5B**). We were not able to observe increased Th17 differentiation in our T cell differentiation assay even in the presence of an increased concentration of IL23. This not surprising as Th17 differentiation require both IL-6 and TGFβ (Veldhoen et al., 2006) and we did not observe any differential expression of TGFβ at mRNA level in our monocyte experiments although is possible that TGFβ is regulated at the translational level. Induction of IL23 will, however, also activate a subset of so-called Type 17 cells which do not require TGFβ (Gaffen et al., 2014). IL23 could then provide two different paths to Type 17 immunity *in vivo* that would not be detected by our *in vitro* assay. The role of INFβ in bacterial infections are, however, still unclear, with evidence pointing to pathogen-specific roles that can be both beneficial and detrimental to the host (Eshleman and Lenz, 2014; Castiglia et al., 2016).

The observed changes in expression of some of the classical macrophage differentiation marker genes (**Supplementary Figure S4**) might indicate the start of monocyte/macrophage reprogramming, however, previous studies demonstrated that many of the underlying transcriptional changes occur at later time-points (Martinez et al., 2006).

**FIGURE 6:**
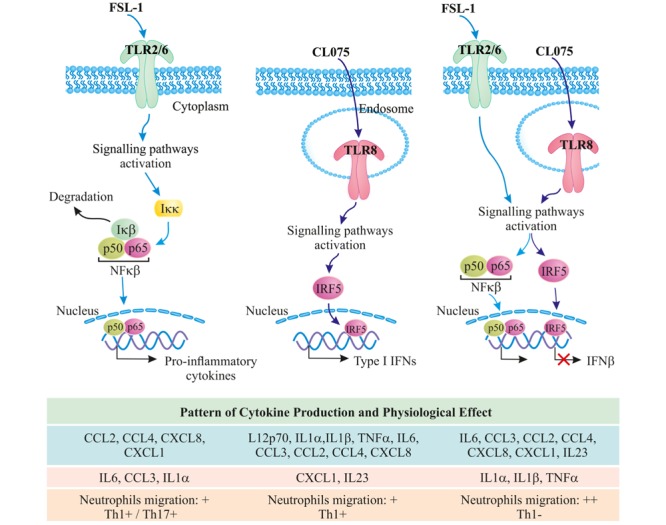
TLR8/TLR2 co-stimulation increases the acute inflammatory response and provides a shift away from Th1 immunity toward increased Th17 immunity; further increasing neutrophil migration.

In total, our gene expression analysis and cytokine profiling predict that co-signaling of TLR8 and TLR2 should increase the acute inflammatory response and provide a shift away from Th1 immunity toward increased Th17 immunity. The observed increase in neutrophil migration and decrease in Th1 differentiation upon co-stimulation underlines this hypothesis (**Figure 6**). We can, however, not explain all the specific mechanisms behind this phenomenon, neither explain whether it is specific to tailor the immune response against pathogens that can stimulate TLR8 and TLR2 simultaneously or simply acts of interference in a complicated signaling environment. It is certainly of interest to note that a Gram-positive bacterial infection should lead to an enhanced neutrophil-based immune response through this mechanism, although it should be noted that most Gram-positive bacteria contain ligands for several other innate immune receptors leading to an even more complex immune signaling pattern. This could also be a mechanism through which monocytes sense an ongoing infection, as live or growing bacteria would contain more TLR8 ligands but the same amount of TLR2 ligands compared to a dead or stationary-phase bacteria (Sander et al., 2011). We also note that while not much is known about IRF5 and IL23 induction, both are heavily involved in autoimmune diseases and dysregulation of either one substantially increases the risk of an overzealous immune response (Eames et al., 2016; Pfeifle et al., 2017). That TLR2 signaling could be able to modulate both of these pathways, at least in human monocytes, might then present a possible target for clinical intervention in both infectious and autoimmune diseases.

## Author Contributions

BB, MG, MH, and MB performed the experiments. BB conceived the study. KB, BB, MG, and MH analyzed the data. BB, MG, MH, and TE designed the experiments. RKK supervised the bioinformatics analysis and follow-up validations. RKK, BB, KB, and MG wrote the manuscript. All authors read and approved the manuscript.

## Conflict of Interest Statement

The authors declare that the research was conducted in the absence of any commercial or financial relationships that could be construed as a potential conflict of interest.

## Supplementary Material

FIGURE S1 **(A)** Measurement of cell surface makers and potential contaminating cell types by Flow-cytometry. Adhered monocytes were detached and subsequently stained with fluorescence-labeled monoclonal antibodies to CD14 and CD11b to identify monocytes, as well as antibodies to CD3, CD19, CD56, and CD303 to identify contaminating cell types. Analysis in the plots was performed on ungated, viable singlet cells. **(B)** mRNA expression of cell type marker genes in samples used for the Array-based RNA expression analysis.

FIGURE S2 Viability analysis of isolated monocytes. Monocytes were stimulated for 3 h with FSL-1, CL075 or both before staining with calcein-AM and propidium iodide. **(A)** Viability analysis by flow-cytometry. Dead cells can be seen in the upper left quadrant of the plots. **(B)** Viability analysis by fluorescence microscopy. Live cells in green, dead cells in red; scale bars 200 μm.

FIGURE S3 Transcriptional changes were measured using Illumina HT-12 v4 bead array. Corresponding volcano plots of expression profiles from CL075, FSL-1 and FSL-1+CL075 stimulated monocytes for 1, 2, and 3 h show that TLR8 stimulation gives a larger magnitude of differentially expressed genes than TLR2 stimulation alone or co-stimulation compared to unstimulated samples at the same time-point.

FIGURE S4 mRNA expression of macrophage differentiation marker genes from CL075, FSL-1 and FSL-1+CL075 stimulated monocytes for 1, 2, and 3 h. CCR5, FCGR1 and CD68 are unaffected, while CD14 and MERTK show a down-regulation; CD40 and CD71 (TFRC) show an up-regulation after 2 h.

FIGURE S5 Enriched Gene Ontology “Biological Processes” terms from differential expressed genes 1, 2, and 3 h CL075, FSL-1 and FSL-1+CL075 stimulated monocytes, manually curated selection.

FIGURE S6 Neutrophils migration toward medium (RPMI+10% serum), 100 ng/mL FSL-1, 1 μg/mL CL07 or combination of both, C5a 10 nM or IL8 10 nM. Neutrophils did not migrate toward TLR2 or TLR8 agonist alone or in combination while IL8 and C5a, two well-known chemotactic factors, significantly increased it. TLR2/8 ligands themselves does not have any significant effect on the neutrophil migration.

FIGURE S7 Clustering and magnitude of mRNA expression11 of phosphatases from primary human monocytes stimulated with FSL-1, CL075 and FSL1+CL075 as compared to non-stimulated samples as log2 fold change. Hierarchical clustering using Manhattan distance matrix calculation was used to divide the expression matrix into 5 clusters. Genes in cluster 1, 3–5 are induced later than genes in Cluster 2. Cluster 2 includes DUSP1 and DUSP2 and which are induced immediately. Cluster 1 contains DUSP3, 4, 8, and 10 and which are induced in a secondary response. Cluster 3 and 5 show only weak changes in expression upon stimulation. Cluster 4 shows down regulation of PPM1M and CTDSP2 in the secondary response.

TABLE S1 List of differentially regulated genes that was used for the clustering analysis.

TABLE S2 List of significantly enriched gene ontology biological processes for the gene clusters.

TABLE S3 *p*-values and significance levels for differential cytokine secretion after TLR8, TLR2 and co-stimulation.
