# Supplementary figures and images for: Coactivation of TLR2 and TLR8 in Primary Human Monocytes Triggers a Distinct Inflammatory Signaling Response
Source: Front Physiol. 2018 May 29;9:618. doi: 10.3389/fphys.2018.00618 (PMC5986927; doi:10.3389/fphys.2018.00618)

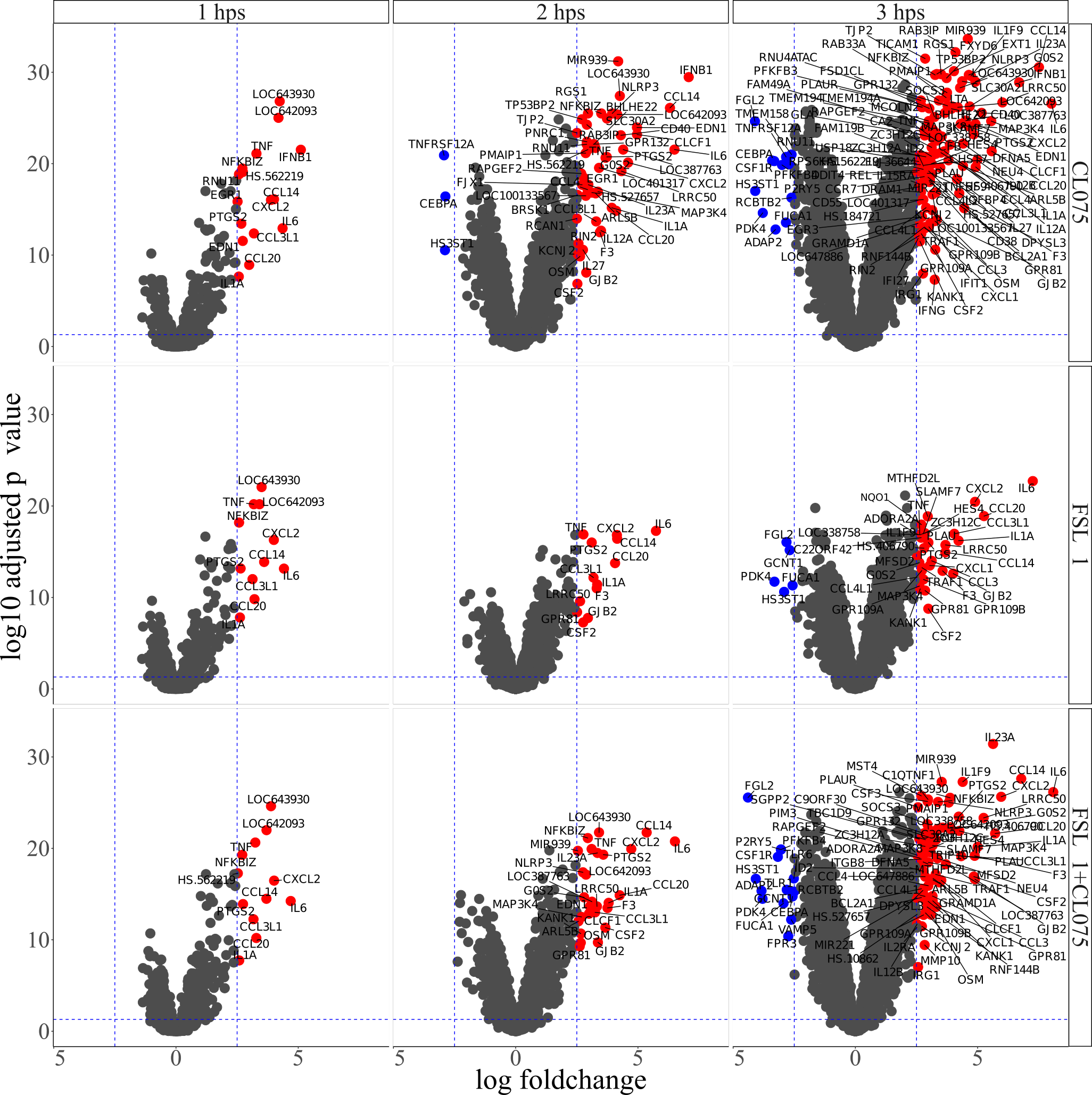

Supplement: FIGURE S3 — Transcriptional changes were measured using Illumina HT-12 v4 bead array. Corresponding volcano plots of expression profiles from CL075, FSL-1 and FSL-1+CL075 stimulated monocytes for 1, 2, and 3 h show that TLR8 stimulation gives a larger magnitude of differentially expressed genes than TLR2 stimulation alone or co-stimulation compared to unstimulated samples at the same time-point. [file Image_3.tiff]
